# Decreased quality of care for *Staphylococcus aureus* bacteremia during the COVID-19 pandemic

**DOI:** 10.1186/s12879-022-07607-9

**Published:** 2022-07-19

**Authors:** Simona Arientová, Zdeněk Jícha, Ondřej Beran, Michal Holub

**Affiliations:** 1 Department of Infectious Diseases, First Faculty of Medicine, Charles University and Military University Hospital Prague, U Vojenské nemocnice 1200, 169 02 Prague 6, Czech Republic; 2 Department of Orthopedics, First Faculty of Medicine, Charles University and Military University Hospital Prague, Prague, Czech Republic

**Keywords:** *Staphylococcus aureus*, Bacteremia, Quality of health care, COVID-19

## Abstract

**Objectives:**

*Staphylococcus aureus* bacteremia (SAB) is one of the most frequent bloodstream infections. High mortality of SAB can be significantly reduced by regular infectious disease (ID) consultations and appropriate clinical management. Because the pandemic of coronavirus disease 2019 (COVID-19) has had a negative impact on hospital ID service, it can be assumed that it has also led to decreased quality of care for SAB patients.

**Methods:**

This study enrolled all (n = 68) patients with proven SAB who were hospitalized in Military University Hospital, Prague, in 2019 and 2020 and the quality of care indicators for SAB patients were compared.

**Results:**

A total of 33 and 35 patients with SAB were hospitalized in our hospital in 2019 and 2020, respectively. The significant difference between the pandemic year 2020 and year 2019 was in ID consultations performed (74% vs. 100%; p = 0.002) and fulfilment of all quality of care indicators (66% vs. 93%; p = 0.012). Next, higher in-hospital mortality was observed in 2020 than in 2019 (6% vs. 23%; p = 0.085). There was no significant difference in the percentages of patients with performed echocardiographic examinations (66% vs. 83%; p = 0.156) and collected follow-up blood cultures (85% vs. 94%; p = 0.428). In addition, there was no difference between the two years in the adequate antibiotic therapy, sources, and bacterial origin of SAB.

**Conclusions:**

The quality of care of SAB patients significantly decreased during the COVID-19 pandemic in our institution.

## Introduction

Bacteremia caused by *Staphylococcus aureus* (SAB) is a serious and frequently fatal condition associated with high incidence and significant mortality [1, 2]. It was repeatedly demonstrated that the care of SAB patients organized by infectious disease (ID) consultants leads to better survival [1, 3–6]. Therefore, the diagnosis of SAB must be followed by appropriate diagnostic and therapeutic processes. Importantly, the quality of care for SAB patients should be assessed using the following indicators: identification of the primary source of infection (such as skin and soft tissue infections, catheter-related infections, infectious endocarditis, osteomyelitis, etc.), early source control, follow-up blood cultures, echocardiography findings, initial and follow-up ID consultations, and appropriate choice and duration of antibiotic treatment [2, 7].

In our tertiary care hospital with 530 acute care beds, the proper management of SAB was a long-term goal, and since 2019, we have been following institutional guidelines. However, as with other hospitals, we were affected by the coronavirus disease 2019 (COVID-19) pandemic in 2020. The pandemic has had a substantial impact on the quality of hospital care and especially on ID specialists that have been significantly involved in the care of COVID-19 patients. Thus, we were interested in how the pandemic affected the quality of care of SAB patients in our institution, and therefore, we compared the quality of care indicators (QCIs) of SAB in the year before the pandemic with those in 2020.

## Materials and methods

### Study cohort

This study enrolled adult patients with proven SAB who were hospitalized in Military University Hospital, Prague, from 1 January 2019 to 31 December 2020. In all patients, *S. aureus* was detected by blood cultures performed during hospitalization.

### Laboratory analysis

All blood culture examinations were performed at the Department of Clinical Microbiology of our hospital. The laboratory utilized BacT/Alert devices (bioMérieux SA, Marcy L'Etoile, France) and haemoculture vials from the same manufacturer. The laboratory used ready-made media (Bio-Rad, California, USA) to cultivate positive samples and determine susceptibility to antibiotics. Identification of the grown cultures was performed with either a mass spectrometer (Bruker, Massachusetts, USA) or a Vitek 2 analyser (bioMérieux SA, Marcy L'Etoile, France).

### Quality of care indicators

The quality of care for SAB patients was assessed using the following indicators:

1. ID bedside consultations.
2. Early source control, including removal of intravascular devices or drainage of infected fluid collection.
3. Follow-up blood cultures 48–96 h after the use of targeted antibiotic therapy.
4. Echocardiography examination was indicated in all patients who had complicated SAB, positive follow-up blood cultures, a high risk of infective endocarditis, or an unclear source of infection. In contrast, in patients with a clear catheter infection and with a negative follow-up blood culture, echocardiography examination was not always required [7].
5. Appropriate choice and duration of antibiotic treatment. For methicillin-sensitive *S. aureus* (MSSA), appropriate antibiotics included oxacillin/flucloxacillin or cefazolin; for methicillin-resistant *S. aureus* (MRSA) it was vancomycin [3, 8]. In the case of bacterial coinfection or superinfection, other β-lactams (cefalosporines, amoxicillin-clavulanate and meropenem) were also accepted according to their sensitivity. Antibiotic dosing was controlled in each patient by clinical pharmacologists, including monitoring of antibiotic serum levels.

For antibiotic therapy to be considered appropriate, it had to be administered intravenously, and duration was defined as ≥ 14 days for uncomplicated SAB and ≥ 28 days for complicated SAB [3, 8]. SAB was considered complicated when infective endocarditis, metastatic spread or prostheses and foreign materials were present and when follow-up blood cultures collected 48 to 96 h after initiation of targeted antibiotic therapy were positive.

To meet the QCIs, it was necessary to fulfil all of the above. From the overall evaluation of QCI, we excluded patients who died within 96 h of admission to the hospital or patients who were transferred to another hospital.

### Statistical analysis

All statistical analyses were performed by a certified biostatistician. Statistica® 12 (StatSoft, TIBCO Software Inc., Palo Alto, CA, USA) software was used. Differences in the values of the variables in the groups were analysed using the T test, Fisher's exact test for count data and Wilcoxon two-sample test, and α < 0.05 was used to define significance. The results are presented as an absolute value with the mean or as the median and range.

## Results

A total of 33 and 35 patients with SAB were hospitalized in our hospital in 2019 and 2020, respectively. Their demographic and clinical characteristics, including in-hospital mortality and sources of infection, are shown in Table 1. In 2020, 2 out of 35 patients with SAB were positive for SARS-CoV-2 by PCR. One of these two patients died after six days in the hospital.

**Table 1:** Demographic, epidemiological, and clinical data of patients with *Staphylococcus aureus* bacteremia

	2019 (n = 33)	2020 (n = 35)	p value
Age (years)*	66 (70; 32–86)	67 (70; 35–97)	0.753
Men, *n* (%)	21 (64)	24 (69)	0.799
DH (days)^*^	23 (22; 2–180)	23 (20; 2–180)	0.394
MSSA/MRSA (%)	88/12	86/14	1
CCI (points)*	6.4 (6; 0–16)	5.3 (6; 0–12)	0.203
In-hospital mortality, *n* (%)	2/33 (6)	8/35 (23)	0.085
MSSA, *n* (%)**	0/2 (0)	6/8 (75)	n.d
MRSA, *n* (%)**	2/2 (100)	2/8 (25)	n.d
Source of infection			
Catheter-related, *n* (%)	11 (33)	10 (29)	n.d
Skin and soft tissue, *n* (%)	8 (24)	6 (17)	n.d
Lung, *n* (%)	5 (15)	7 (20)	n.d
Bone and joint, *n* (%)	5 (15)	6 (17)	n.d
Infectious endocarditis, *n* (%)	3 (10)	4 (11)	n.d
Urinary tract, *n* (%)	0	1 (3)	n.d
Unidentified, *n* (%)	1 (3)	1 (3)	n.d

*Data are expressed as the means (medians; ranges); **Data of patients succumbed to SAB only; *DH* duration of hospitalization; *MSSA* methicillin-sensitive *S. aureus*; *MRSA* methicillin-resistant *S. aureus*; *CCI* Charlson Comorbidity Index; *n.d* not defined

The QCIs of SAB obtained for both evaluated years are shown in Table 2.

**Table 2:** Comparison of quality of care indicators between the groups

Quality of care indicators	2019 (n = 33)	2020 (n = 35)	p value
ID consultation, *n* (%)	33/33 (100)	26/35 (74)	0.002
Early source control, *n* (%)			
Removal of intravascular catheter^a^	11/11 (100)	10/10 (100)	n.d
Surgical intervention/drainage^b^	4/4 (100)	2/2 (100)	n.d
Follow-up blood culture, *n* (%)	29/31* (94)	28/33* (85)	0.428
Echocardiography, *n* (%)	25/30* (83)	23/33* (70)	0.156
TTE, *n* (%)	16/30* (53)	17/33* (52)	n.d
TEE, *n* (%)	9/30* (30)	6/33* (18)	n.d
Adequate antibiotic therapy, *n* (%)	28/29* (97)	30/31* (97)	1
Uncomplicated SAB, *n* (%)	9/33 (27)	11/35 (31)	n.d
Complicated SAB, *n* (%)	24/33 (73)	24/35 (69)	n.d
Fulfillment of indicators, *n* (%)	27/29* (93)	21/32* (66)*	0.012

^a^Patient with a catheter infection; ^b^patient with a soft tissue abscess or surgical wound abscess; *Individual quality indicators were not determined in all patients due to the early death of the patients or transfer to another hospital; *ID* infectious disease; *SAB* *Staphylococcus aureus* bacteremia; *TTE* transthoracic echocardiography; *TEE* transesophageal echocardiography; *n.d* not defined

Regarding antibiotic therapy for SAB caused by MSSA, 83% and 63% of the patients were switched to targeted oxacillin therapy with an appropriate dose and duration of treatment in 2019 and 2020, respectively. A total of 17% of patients in 2019 and 37% in 2020 were treated with other antibiotics with appropriate doses and durations of therapy. In all MRSA-related SAB patients, vancomycin therapy was correctly used in both years. Out of all the patients in both years, only one patient (3%) was treated with antibiotics for less than 14 days.

Because the pandemic year 2020 was associated with a significant decrease in ID consultations, we compared the QCIs in patients seen and not seen by ID consultant. Interestingly, the patients without ID consultation had significantly lower percentages of properly collected blood cultures (70% vs. 91%), performed echocardiographic examinations (40% vs. 90%) and fulfilled QCIs (30% vs. 82%). In contrast, there were minimal differences in antibiotic therapy (95% vs. 100%) and mortality (25% vs. 22%).

In addition, 17% of SAB patients (6 of 35) in 2020 were hospitalized before the advent of the COVID-19 pandemic, and all of them survived.

## Discussion

In the present study comparing the quality of care between 2019 and 2020, we observed a negative impact of the COVID-19 pandemic on the appropriate management of SAB in our tertiary care facility.

Importantly, we observed a four times higher number of deaths among SAB patients in 2020 than in 2019 (prepandemic). Although in-hospital mortality in 2020 was not significantly different from that in 2019, it returned to the same level as in the period before the introduction of any intervention in our hospital, 28% in 2015 and 27% in 2016 [9]. In 2017 and 2018, we started to implement the principles of SAB management, with annual in-hospital mortality rates of 16% and 18%, respectively. However, in this period, ID consultations were mostly unsolicited and associated with low adherence. Similar differences in SAB-related mortality were reported before and after the introduction of proper management in several studies: Bai et al. [8] described a reduction in patient in-hospital mortality from 29 to 21%, López-Cortés et al. [10] from 18 to 11% (14-day mortality) and Vogel et al. [2] from 26 to 12% (30-day mortality). The more deaths from SAB observed in our facility in 2020 could have been related to the COVID-19 pandemic because ID specialists had heavy workloads that included providing consultations in all specialized COVID-19 wards (60 beds) and ICUs (22 beds) and directly taking care of oxygen-dependent COVID-19 patients in the ID department (27 beds). This is further supported by the fact that ID consultations were performed during the pandemic year in a significantly lower percentage of SAB patients than in 2019. It is probable that less frequent ID consultations could lead to poorer compliance with the SAB institutional guidelines because several studies have demonstrated that the ID consultation at the bedside is associated with better SAB management and a significant reduction in mortality [1, 3, 7]. In addition, the clinical characteristics of SAB patients were not found to significantly influence their outcomes. However, it is important to consider a potential role of some unmeasured confounding factors.

When we evaluated the overall quality of care in both years by evaluating the monitored QCIs, we found that the procedures were followed for all but two patients in 2019, compared with only 2/3 of the patients in 2020. Similar differences were reported by other authors who compared periods before and after the implementation of SAB institutional guidelines [2, 8, 10, 11]. However, to our knowledge, the direct impact of the COVID-19 pandemic on the quality of care of SAB patients has not been reported in the literature, and only a few studies have evaluated the impact of the COVID-19 pandemic on SAB. Weiner-Lastinger et al. described a higher incidence of MRSA-induced catheter bacteremia in 2020 than in 2019 [12], and Baker et al. reported increases in catheter-related infection of 60% and MRSA bacteremia of 44% [13]. However, these reports are in contrast with the situation in our facility because the incidence of SAB did not differ between prepandemic and pandemic years.

It is also very important to choose the correct antibiotic therapy for SAB: the first drug of choice for MSSA infections is intravenous oxacillin/cloxacillin and vancomycin for MRSA infection [14]. However, it is no less important to treat SAB with an appropriate duration of antibiotic therapy, which depends on whether the infection is complicated or uncomplicated. Complicated SAB must then be treated with antibiotics for at least 28 days (6 weeks in the case of infectious endocarditis), whereas uncomplicated infection is usually treated for 14 days [1, 8]. In the context of antimicrobial therapy for SAB, it is necessary to stress that 97% of all evaluated patients in both years were treated with appropriate antibiotics for an adequate duration, indicating that the factors related to antimicrobial therapy were not compromised in our facility during the COVID-19 pandemic. The reason is probably the fact that antibiotic therapy was always discussed with the antibiotic centre in collaboration with microbiologists and ID specialists. Moreover, the monitoring of antibiotic levels, including their toxicity, was performed by a clinical pharmacist, which is the standard of care in tertiary care hospitals in the Czech Republic.

## Conclusion

The data from the first year of the COVID-19 pandemic demonstrated significant alterations in the quality of care for SAB patients in comparison to 2019 (prepandemic). Because the pandemic had an especially strong negative impact on our hospital ID service, the data may support its importance in the management of SAB.

## Data Availability

All data generated or analysed during this study are included in this published article.

## Abbreviations

COVID-19: Coronavirus disease 2019
ID: Infectious disease
MRSA: Methicillin-resistant *S. aureus*
MSSA: Methicillin-sensitive *S. aureus*
PCR SARS-CoV-2: Polymerase chain reaction of severe acute respiratory syndrome coronavirus 2
QCIs: The quality of care indicators
SAB: *Staphylococcus aureus* Bacteremia
